# Does intensive management improve remission rates in patients with intermediate rheumatoid arthritis? (the TITRATE trial): study protocol for a randomised controlled trial

**DOI:** 10.1186/s13063-017-2330-8

**Published:** 2017-12-08

**Authors:** Naomi H. Martin, Fowzia Ibrahim, Brian Tom, James Galloway, Allan Wailoo, Jonathan Tosh, Heidi Lempp, Louise Prothero, Sofia Georgopoulou, Jackie Sturt, David L. Scott, On behalf of TITRATE Programme Investigators, Heidi Lempp, Jackie Sturt, Sofia Georgopoulou, Louise Prothero, Naomi Martin, Richard Jenner, Isabel Neatrour, Rhiannon Baggott, Fowzia Ibrahim, Brian Tom, Allan Wailoo, Jonathan Tosh, James Galloway, Gabrielle Kingsley, David Scott, Brian Tom, Fowzia Ibrahim, Yujie Zhong, Aneela Mian, James Galloway, David L. Scott

**Affiliations:** 1 Academic Department of Rheumatology, King’s College London, Weston Education Centre, Cutcombe Road, Denmark Hill, London, SE5 9RJ UK; 2 MRC Biostatistics Unit, Institute of Public Health, University Forvie Site, Robinson Way, Cambridge, CB2 0SR UK; 3 Health Economics and Decision Science, School of Health and Related Research, University of Sheffield, Regent Court, 30 Regent Street, Sheffield, S1 4DA UK; 4 DRG Abacus, Manchester One, 53 Portland Street, Manchester, M1 3LF UK; 5 Department of Physiotherapy, King’s College London, 5th Floor, Addison House, Guy’s Campus, London, SE1 1UL UK; 6 Florence Nightingale Faculty of Nursing and Midwifery, King’s College London, James Clerk Maxwell Building, 57 Waterloo Road, London, SE1 8WA UK

**Keywords:** Rheumatoid arthritis, Intermediate disease activity, Treating to target, Intensive treatment, Randomised controlled trial, Tumour necrosis factor inhibitors

## Abstract

**Background:**

Uncontrolled active rheumatoid arthritis can lead to increasing disability and reduced quality of life over time. ‘Treating to target’ has been shown to be effective in active established disease and also in early disease. However, there is a lack of nationally agreed treatment protocols for patients with established rheumatoid arthritis who have intermediate disease activity. This trial is designed to investigate whether intensive management of disease leads to a greater number of remissions at 12 months. Levels of disability and quality of life, and acceptability and cost-effectiveness of the intervention will also be examined.

**Methods:**

The trial is a 12-month, pragmatic, randomised, open-label, two-arm, parallel-group, multicentre trial undertaken at specialist rheumatology centres across England. Three hundred and ninety-eight patients with established rheumatoid arthritis will be recruited. They will currently have intermediate disease activity (disease activity score for 28 joints assessed using an erythrocyte sedimentation rate of 3.2 to 5.1 with at least three active joints) and will be taking at least one disease-modifying anti-rheumatic drug.

Participants will be randomly selected to receive intensive management or standard care. Intensive management will involve monthly clinical reviews with a specialist health practitioner, where drug treatment will be optimised and an individualised treatment support programme delivered based on several principles of motivational interviewing to address identified problem areas, such as pain, fatigue and adherence. Standard care will follow standard local pathways and will be in line with current English guidelines from the National Institute for Health and Clinical Excellence. Patients will be assessed initially and at 6 and 12 months through self-completed questionnaires and clinical evaluation.

**Discussion:**

The trial will establish whether the known benefits of intensive treatment strategies in active rheumatoid arthritis are also seen in patients with established rheumatoid arthritis who have moderately active disease. It will evaluate both the clinical and cost-effectiveness of intensive treatment.

**Trial registration:**

Current Controlled Trials, ID: ISRCTN70160382. Registered on 16 January 2014.

**Electronic supplementary material:**

The online version of this article (doi:10.1186/s13063-017-2330-8) contains supplementary material, which is available to authorized users.

## Background

Rheumatoid arthritis (RA) is an immunologically driven, progressive, long-term condition. It is characterised by persistent synovitis, systemic inflammation and detectable autoantibodies, including rheumatoid factor and anti-cyclic citrullinated peptide antibody [1]. Ongoing joint inflammation damages cartilage, bone and tendons; systemic inflammation causes extra-articular complications like vasculitis and lung disease. Uncontrolled active RA leads to disability, decreased quality of life (QoL) and increased co-morbidity. The end result is loss of work, major medical and social costs and high morbidity and mortality [2, 3].

RA management involves a multidisciplinary team including rheumatologists, specialist nurses, therapists and others. The team provide education, particularly on self-management, medication, psychological support, exercise and joint protection [4]. RA outcomes are optimised by treating patients to pre-defined targets [5–7]; the most appropriate target is remission.

Drug treatment focusses on controlling joint inflammation with disease-modifying anti-rheumatic drugs (DMARDs). They reduce synovitis, systemic inflammation and disability. The dominant DMARD is methotrexate; others include sulfasalazine and leflunomide [8]. The impact of DMARDs can be maximised by using them in combination. However, side effects limit DMARD use by both clinicians and patients [4]. Steroids (glucocorticoids) also reduce joint inflammation [9]. In the short-term steroids can be combined with DMARDs to reduce erosions and to treat systemic disease. Long-term steroid use has unacceptable toxicity [10].

Biological agents, given when DMARDs cannot control RA, have revolutionised its management. Biologics such as tumour necrosis factor inhibitors (TNFi), rituximab, abatacept and tocilizumab are highly effective [11]. They are mainly given with methotrexate to increase efficacy and reduce blocking antibodies [12]. Their main risk is infection [13] and use is limited by their high costs [14]. Although they substantially improve RA outcome, they do not cure the disease.

RA patients are distinguished into categories according to their disease activity levels. This is currently undertaken on the basis of the Disease Activity Score for 28 joints (DAS28), which is a composite measure including assessment of tender and swollen joint (based on 28 joints), the erythrocyte sedimentation rate (ESR) and patient global assessments on a 100-mm Visual Analogue Scale (VAS) [15].

DAS28 scores divide patients with established RA into four categories. These are as follows:

1. High disease activity (DAS28 over 5.1)
2. Intermediate (or moderate) disease activity (DAS28 over 3.2 to 5.1)
3. Low disease activity (DAS28 2.6 to 3.2)
4. Remission (DAS28 under 2.6)

Randomised clinical trials (RCTs) enrol patients with RA who have high disease activity. There is strong evidence from RCTs that patients with RA with high disease activity benefit from treatment with DMARDs and biologics. Remission or low disease activity are the goals of treatment. This approach, termed ‘treat to target’ [16], is supported by a strong evidence base [17, 18]. When patients achieve sustained low disease activity or remission, most clinicians either maintain treatment or reduce treatment levels. However, many RA patients currently attending rheumatology clinics have intermediate (or moderate) disease activity levels [19]. There is only limited evidence that intensive treatment strategies benefit such patients and there is uncertainty about how best to treat them. One key reason for these doubts is that such patients are not usually included within RCTs [19–22].

There are a number of clinical guidelines that summarise how best to manage patients with RA [23–25]. Those by the National Institute for Health and Clinical Excellence (NICE) are the most relevant for clinical practice in England [25]. They make some general recommendations on the current management of patients with intermediate disease, which can be summarised as follows:

- Maintain suppressive treatment with DMARDs and steroids
- Maintain symptomatic therapy (analgesics/non-steroidal anti-inflammatory drugs)
- Carry out annual specialist reviews, with urgent specialist reviews and treatment modification for flares (DAS28 over 5.1) or clinically significant adverse events

There has been discussion about the relative merits of giving treatments, such as biologics, to patients with intermediate disease activity, but no nationally agreed protocols exist in England. There is substantial international variation on the use of biological treatments and in some European countries and in North America, patients with intermediate disease activity are often prescribed biological treatment. The absence of any agreed treatment protocols for patients with intermediate disease activity is a major challenge in defining the most appropriate way to treat many RA patients attending specialist clinics in England [25].

A number of strategy trials have shown the benefits of combining treatments – DMARDs, steroids and, in some trials, biologics – to optimise outcomes, and have confirmed the benefit of treating to target, where patients are treated until they reach the therapeutic target of remission or low disease activity [16]. ‘Intensive management’ (IM) approaches used in these strategy trials give the best outcomes for RA so far reported [17, 18].

A characteristic feature of RA trials is their focus on increasing treatment in patients with high disease activity. One reason is that such patients have pressing needs to justify treatment change. A second is the relative ease of showing treatment benefit in patients with high disease activity. As a consequence, trials rarely enrol patients with intermediate disease activity [19–22]. A common feature of patients with intermediate disease is the duration of their RA and multiple historical treatment changes that have often been made to achieve acceptable levels of disease activity for the patient. Recruiting intermediate patients to a medication optimisation trial would require additional support components that would result in patients taking medications that they may have perceived to have been previously unsuccessful. Therefore, optimising treatment in intermediate disease requires components that would address knowledge, motivation and shared decision-making [26].

Medication can alleviate symptoms and halt disease progression in rheumatoid arthritis (RA). Nevertheless, medication non-adherence is common in RA and poses a significant barrier to improving clinical outcomes in RA with only 58–82% of RA patients adhering to DMARDs [27]. Factors that can influence adherence include patients’ negative beliefs about medicines and their condition, as well as their degree of satisfaction with information about DMARDs [28, 29]. Similarly, adherence to biologic treatments, such as adalimumab, is driven by psychological factors, particularly medication beliefs [30].

In addition to the impact that RA has on joints and physical disability, it also significantly affects QoL [31]. Fatigue is reported in over 80% of RA patients [32–34] and 57% of RA patients identify fatigue as the most problematic symptom of their condition [35]. Disease activity might not be the sole factor exerting a significant impact on fatigue; it may also result from a constellation of factors that include disease activity or pain, inactivity, depression, obesity and poor sleep [36]. Due to the pervasive effect of RA on patient outcomes and its impact on health status and QoL, it was deemed crucial to incorporate a component of ‘psychosocial support’ in the intervention. ‘Psychosocial support’ aimed to address the challenging domains of coping with RA including medication adherence by influencing behaviour change.

Against this background, the key reasons for undertaking the TITRATE trial are:

- Remission is the most appropriate target in RA [37]
- Intensive management regimens using DMARD combinations, steroids and sometimes biologics, together with a ‘treatment support’ programme of effective non-drug interventions and psychosocial support for coping with the various domains of RA are most likely to achieve remission [38, 39]
- The most important group of patients in which to investigate whether intensive management achieves remission are those with ‘intermediate disease activity’; these patients currently continue to have persisting disease activity and as a consequence they develop progressive disability [40]

The TITRATE trial is designed to show whether patients with intermediate disease activity benefit from such intensive management.

As a consequence of these considerations the trial will enrol patients with intermediately active RA defined by their DAS28 scores. The intervention will be intensive management with DMARDs and biologics given in a supportive manner agreed with individual patients. The control group will receive standard care following existing national guidance. The

primary outcome will be remission at 12 months assessed using DAS28. The hypothesis is that intensive management will increase the number of patients in DAS28 remission at 12 months compared with SC.

## Methods

### Aim and hypothesis

TITRATE is a robust pragmatic clinical trial to improve outcomes for RA patients with intermediate disease activity by using an Intensive Management Programme. The trial will test the hypothesis that patients with established RA, who currently have intermediate disease activity (defined as DAS28-ESR of 3.2–5.1 with at least three active joints) and are currently receiving at least one DMARD, are more likely to achieve remission at 12 months if they receive intensive management than if they continue to have SC.

The primary objective is to improve outcomes defined through achieving remission at 12 months for RA patients with intermediate disease activity using intensive management.

The secondary objectives are as follows:

#### Clinical outcomes

- To assess disability with the Health Assessment Questionnaire (HAQ)
- To determine the relative effect on quality of life (QoL)
- To assess acceptability of intensive management to RA patients with intermediate disease activity
- To assess the risks of adverse events from intensive management

#### Economic outcome

- To determine the cost-effectiveness of intensive management

### Design

**Fig. 1:**
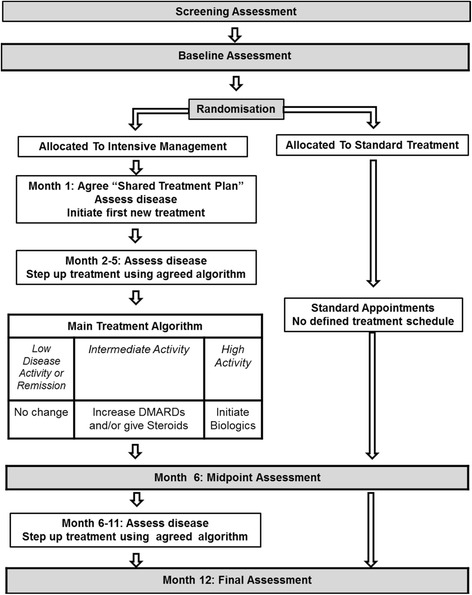
Study design: outline of intensive management approach and standard treatment arms

**Fig. 2:**
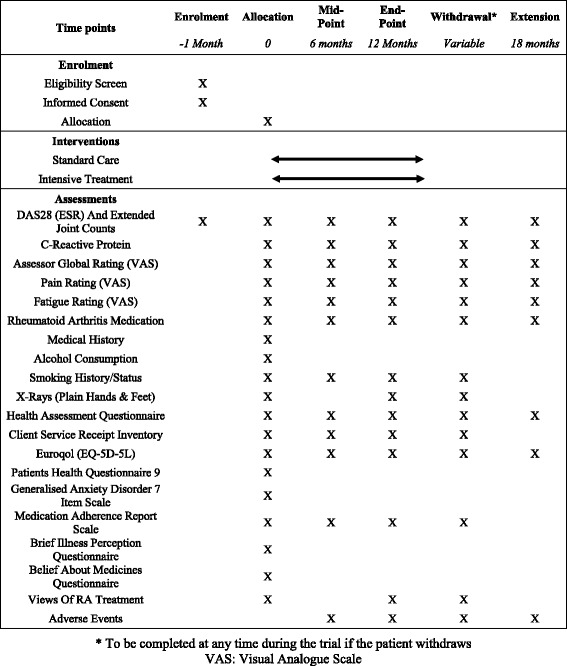
Standard Protocol Items Recommendations for Interventional Trials (SPIRIT) Schedule of enrolment, interventions and assessments

The TITRATE trial is a 12-month, pragmatic, randomised, open-label, two-arm, parallel-group, multicentre trial undertaken at 35–40 specialist rheumatology clinics across England. The trial was designed by key stakeholders including rheumatologists with experience of treating RA, specialist nurses, GPs with a special interest in musculoskeletal disease, methodologists and two service users, who were patients with personal experiences of living with RA. The study design outlining the intensive management approach and standard treatment is shown in Fig. 1. A Standard Protocol Items: Recommendations for Interventional Trials (SPIRIT) Checklist is provided as Additional file 1, and a flow diagram is included as Fig. 2.

The trial assesses a treatment strategy rather than any particular drug therapy and all treatments will be used within their current marketing authorisation. Consequently, the Medicines and Healthcare Products Regulatory Agency decided that the trial did not meet their criteria for a Clinical Trial of an Investigational Medicinal Product (CTIMP) and that TITRATE was a non-CTIMP trial.

Ethical approval for this trial was obtained from the London – West London and GTAC National Research Ethics Service (NRES) Committee.

### Setting

The trial will be undertaken within routine rheumatology outpatient clinics in approximately 40 hospitals in England. A full list of participating centres is available at https://www.kcl.ac.uk/lsm/research/divisions/diiid/departments/rheumatology/research/clinical/current/titrate/introduction.aspx.

### Target population

The target population will be patients attending specialist rheumatology clinics who meet the most recent classification criteria for RA (American College of Rheumatology 2010 criteria); have established RA; currently have intermediate disease activity (DAS28-ESR of 3.2–5.1 and at least three active joints) and are receiving at least one conventional DMARD. Participants will need to meet the following eligibility criteria:

#### Inclusion criteria

1. Men and women aged over 18 years
2. Diagnosis of RA (by American College of Rheumatology (ACR), 2010 criteria) [41]
3. Have received at least one DMARD for at least 6 months, and currently receiving at least one DMARD
4. Have intermediate disease activity, defined by: (a) DAS28-ESR of 3.2–5.1; (b) at least three active joints (defined as swollen and/or tender) on 66/68 joint count, to include at least one swollen joint
5. Willing and able to follow an Intensive Management Programme
6. Able and willing to give informed consent

#### Exclusion criteria

1. Major co-morbidities making intensive treatment inadvisable (e.g. heart failure)
2. Previously failed multiple DMARDs (at least five treatments) or having received biologics
3. Irreversible disability from extensive joint damage (e.g. replacement of three or more major joints)
4. Women who are pregnant, breast-feeding or at risk of conceiving
5. Current or recent (within the 12 weeks prior to randomisation) participation in another interventional trial
6. Currently in an early RA pathway, which is a 12-month treatment programme for patients with early inflammatory arthritis, in which patients receive intensive treatment with DMARD combinations and steroids and are reviewed monthly by specialist nurses or equivalent members of the rheumatology team; this is one of the current NHS Best Practice Tariffs [42]

### Interventions

#### Standard care

In the standard care arm, clinicians will follow their local pathways for managing RA patients with intermediate disease activity. These will be based on national guidance from NICE. The key components of the standard care algorithm comprise:

- Maintaining suppressive treatment with DMARDs and steroids
- Seeing patients at least once each year in line with local pathways of care
- Arranging urgent specialist reviews using routine approaches if there be a clinical need

#### Intensive management

In the intensive management arm, patients will be seen monthly by trained health practitioners, who will often, but not always, be a nurse identified by the principal investigator as being competent to provide the intervention, who will:

- Assess their RA and current general functioning
- Evaluate their drug treatment
- Modify the drug treatment according to a decision tool (or algorithm) in line with a ‘shared treatment plan’ formulated during the first visit. Shared treatment plans will involve agreements with patients about drugs, dosages and therapeutic sequences. Templates for potential shared treatment plans were developed with patients in advance of the trial as part of a preparatory study within the TITRATE programme
- Provide supportive care according to the principles, knowledge and skills acquired in the training course which are underpinned by Motivational Interviewing techniques and are included in the Treatment Support Manual

The Intensive Management Programme will address the following:

1. Provide information: there will be a handbook for patients [43] which will describe in detail the various aspects of intensive treatment as well as information on psychoeducation such as details of medication side effects, ways of coping with the physical and emotional symptoms and the impact of RA on everyday life, contact details for support groups, and disability information
2. Optimise DMARDs/biologics: drug treatment will be modified following a treatment algorithm, which will recommend treatment options based on previous treatment, present treatment, contraindications, the patient’s preferences and clinical assessments. The premise of the treatment algorithm is that, if the patient’s disease is still active, whatever the current treatment strategy, the recommended strategy will always reflect potential intensification of treatment. All medication given to patients in the intensive management group will be in line with national guidance from NICE or the national specialist society (British Society for Rheumatology) [44] the only differences being that patients will be reviewed more frequently than they would be under standard care and if their disease is not fully controlled may be given biological therapies in line with British Society for Rheumatology recommendations. Local safety screening for tuberculosis and other infections will be performed according to local guidelines for patients in the intensive management arm prior to starting biologics (if needed) in line with British Society for Rheumatology guidance
3. Give steroids: patients will receive intra-muscularly administered steroids (depomedrone or equivalent) if their arthritis is not fully controlled up to a maximum of 600 mg depomedrone (or equivalent) over the 12-month period. The dose of steroids given will range from 40 to 120 mg depending on specific clinical circumstances
4. Provide treatment support: along with treating patients more intensively with medication, the trained rheumatology practitioner will also provide patients in this group with supportive care. Patients will be educated and supported in a number of domains commonly affected by RA, with a particular focus on: pain and fatigue management; physical activity; medication adherence, sleep and low mood/anxiety. Patients will also have a handbook, co-developed with patients and national charities, which includes psychoeducation about their condition, treatment options and ways of coping with the physical and emotional symptoms

All specialist rheumatology practitioners involved in delivering the intensive management intervention will be trained according to a manualised training programme to ensure that the intervention provided is standardised across sites and participants. The training involves a 2-day course, with day 1 focussing solely on motivational interviewing techniques; specifically, open-ended questions, affirmations, reflections/reflective listening and summaries and the treatment algorithm for intensive management. The second day focusses on psychoeducation regarding various aspects of RA including preparation for behaviour change, goal-setting, diary keeping, self-monitoring, development of a shared treatment plan, standardisation of the DAS28 scoring process, explanation of the process of recording the sessions and the supervision that will be available to practitioners [26]. To measure adherence to the intensive management intervention across sites, monthly sessions will be audiotaped and a randomly selected subset of these rated by a team of three independent assessors.

### Assessments

Assessments will be carried out by a member of the research team at each participating centre. Following consent and confirmation of eligibility, all patients will complete an initial baseline assessment. This will be followed by a midpoint and final assessment at 6 and 12 months from baseline, respectively. See Table 1 for a summary of the milestone research assessments.

**Table 1:** Summary of milestone assessments

Assessment	Baseline	Month 6 (midpoint)	Month 12 (final)	Withdrawal^a^	Extension study^b^
DAS28 (ESR) and Extended Joint Count	X	X	X	X	X
CRP	X	X	X	X	X
Assessor global rating (VAS)	X	X	X	X	X
Pain rating (VAS)	X	X	X	X	X
Fatigue rating (VAS)	X	X	X	X	X
RA medication	X	X	X	X	X
Medical history	X				
Alcohol consumption	X				
Smoking history/status	X	X	X	X	
X-rays (plain, of hands and feet)	X		X	X	
HAQ	X	X	X	X	X
CSRI	X	X	X	X	
EQ-5D-5 L	X	X	X	X	X
PHQ-9	X				
GAD-7	X				
MARS	X	X	X	X	
BIPQ	X				
BMQ	X				
Views of RA treatment	X		X	X	
Adverse events		X	X	X	X

DAS28-ESR will be calculated from joint count, patient global rating (VAS) and ESR during every visit; SDAI will be calculated from joint count, patient global rating (VAS), assessor global rating (VAS) and CRP at baseline, midpoint and final assessments

^a^To be completed at any time during the trial if the patient withdraws. ^b^Extension study only

*BIPQ* Brief Illness Perceptions Questionnaire, *BMQ* Beliefs about Medicines Questionnaire, *CRP* C-reactive protein*, CSRI* Modified Client Service Receipt Inventory, *EQ-5D-5 L* EuroQol 5-dimensional 5-level score, *ESR* erythrocyte sedimentation rate, *GAD-7* Generalized Anxiety disorder-7, *HAQ* Health Assessment Questionnaire, *MARS* Medication Adherence Rating Scale, *PHQ-9* Patient Health Questionnaire-9, *RA* rheumatoid arthritis, *SDAI* Simplified Disease Activity Index, *VAS* Visual Analogue Scale

#### Primary outcome measure

The primary outcome will be the number of patients in each treatment arm fulfilling the definition of remission as measured by DAS28-ESR (remission defined as DAS28-ESR < 2.6) [45, 46] at 12 months.

#### Secondary outcome measures

These will assess the following outcomes at 6 and/or 12 months:

- *Alternative assessments of remission*: remission measured by the Disease Activity Score for 28 joints-C-reactive protein score (DAS28-CRP) [46, 47] the Simplified Disease Activity Index (SDAI) (remission defined as SDAI ≤ 3.3) [48] at 12 months; remission assessed by all measures at 6 months
- *Assessment of individual components of remission*: tender joint counts (68 joints), swollen joint counts (66 joints), patient global assessments on 100-mm a Visual Analogue Scale (VAS), assessor global assessments on 100-mm VAS, C-reactive protein (CRP) and erythrocyte sedimentation rate (ESR)
- *Disability*: Health Assessment Questionnaire (HAQ) [49]
- *Joint imaging (predictor of future disability)*: plain X-rays of the hands and feet read by a modified Larsen’s score [50]
- *Quality of life*: EuroQoL 5-dimensional 5-level score (EQ-5D-5 L) [51], fatigue rating (VAS)
- *Patient acceptability*: Modified Measuring Actual Patient-led Expectations in Rheumatoid Arthritis (MAPLe-RA) [52], Medication Adherence Rating Scale (MARS) [53]
- *Adverse events*
- *Economic assessments*: Modified Client Service Receipt Inventory (CSRI) [54]

#### Potential predictor variables (psychosocial measures)

Baseline assessments will include evaluation of a number of potential outcome predictors, which will be used in exploratory analyses. These will include:

- Lifestyle factors: alcohol consumption and tobacco smoking
- Mood and anxiety: Patient Health Questionnaire-9 (PHQ-9) [55] and Generalised Anxiety Disorder-7 (GAD-7) [56]
- Health beliefs/illness perceptions: Beliefs about Medicines Questionnaire (BMQ) [57] and Brief Illness Perceptions Questionnaire (BIPQ) [58]

### Withdrawal of participants

Participants have the right to withdraw from the study at any time for any reason. Patients wishing to withdraw from the study will be asked to complete a withdrawal assessment, which is the equivalent of a final (month-12) assessment.

Patients in the intensive management arm who wish to withdraw will be given the following options:

1. *Withdrawal from intervention*: revert to treatment as usual and complete 6- and/or 12-month follow-up assessments
2. *Medical note review only*: revert to treatment as usual but consent to collection of data from routine medical notes (no follow-up assessments completed)
3. *Withdrawal from research*: refuse any further collection of outcome data, either through follow-up assessments or medical note review

Patients in the standard care arm who wish to withdraw will be given the following options:

1. *Medical note review onl*y: consent to collection of data from routine medical notes (no follow-up assessments completed)
2. *Withdrawal from research*: refuse any further collection of outcome data, either through follow-up assessments or medical note review

### Extension study for patients in the intensive management arm

To investigate whether disease activity and the impact of RA on general health remain stable after a period of 12 months of intensive management, we will undertake an exploratory study of the future outcome of patients in the intensive management arm of the trial. After completing 12 months in the intensive management arm, patients will return to receiving contemporary standard care treatment following local pathways for managing RA patients with intermediate disease activity. These patients will be invited to complete an additional assessment 6 months after completing the trial. This assessment will include measures of remission and a subset of patient-completed questionnaires used in the main trial. See Table 1 for further details.

### Sample size

One of the most relevant UK trials (TICORA) compared tight control versus standard treatment in patients with RA for less than 5 years; it reported that 16% of patients receiving standard care achieved DAS remission at the end of the trial [59]. We therefore assume that with standard care 16% of patients will have achieved DAS remission at 1-year follow-up.

We will reject the null hypothesis (RA patients with intermediate disease activity (DAS28 3.2–5.1), despite DMARDs, will not have more remissions following 12 months of intensive management) if the difference in remission rates at 12 months between the intensive management arm and the standard care arm is 15% or greater. Demonstrating such a difference with 5% significance and 90% power requires randomising 358 patients in total, under 1:1 allocation (i.e. 179 patients per group). However, if we assume that 10% of patients will not provide follow-up information at the end of the study the required total sample size increases to 398 patients (199 per arm).

### Randomisation

Only when all baseline measures are complete and data is entered will patients be randomised. Randomisation will be at the level of the individual using block randomisation with randomly varying block sizes, to ensure pre-randomisation allocation concealment, stratified by site. Patients will be randomised to intensive management (IM) or standard care (SC) in a ratio of 1:1. All staff involved in the conduct of the trial will be unaware of the allocation sequence. The trial will not be blinded. Patients are an integral part of the intensive treatment algorithm and there is no possible way to blind such a trial.

### Data analysis

Analysis will be on an intention-to-treat (ITT) basis to reflect the randomisation process. We will also carry out two additional analyses populations: a complete case population: these will be observations that subjects complete the trial without missing data or violation of the protocol and, therefore, referred to as ‘complete case analysis’. A per-protocol population: these will be observations that will be excluded from these analyses if patients are found to deviate from the protocol and referred to as ‘per-protocol analysis’.

Baseline characteristics will be summarised by randomised group. The Multiple Imputations (MI) method will be used to impute missing primary or secondary outcomes. The robustness of the analyses performed to the missing at random assumption under MI model will be assessed by Linear Increment method of Diggle et al. [60] to handle the missingness.

A logistic regression analysis will be used to analyse the primary outcome – remission at 12 months. For secondary analyses that involve longitudinal measurements, generalised estimating equations (GEE) and/or mixed models will be used to estimate the effect of treatment, including baseline value as a covariate. Working correlation matrices will be unstructured, which is not unduly restrictive given that measurements will be taken at three time points. Valid/robust estimates of the precision of effects will be obtained through use of the information sandwich estimator for GEE analyses.

Treatment, and the demographic factors (age, ethnicity, gender, disease duration) as well as the design factor (region) will be included as explanatory variables in the multivariate analysis. The design factor will also be accounted for in the univariate model. The estimates for primary outcome will be presented as odds ratios (OR) with 95% confidence intervals for the effect of intensive management. Statistical significance will be determined at the 5% level using a two-sided test throughout.

Serious adverse event and adverse rates in the two arms will be compared using comparisons of two independent proportions.

Analysis of the exploratory extension study will use the final assessment in the TITRATE trial and the new 6-month follow-up data to compare the proportion of patients in remission at 12 and 18 months. Simple descriptive analysis will be used to determine the numbers of patients in remission and changes in the key clinical and functional outcomes.

A full statistical analysis plan was developed prior to the start of the trial.

### Cost utility analysis

A cost-utility analysis will be undertaken to estimate the incremental cost per quality-adjusted life year (QALY) of intensive management compared to standard care in RA patients with intermediate disease activity, alongside the clinical trial.

The cost-utility analysis will be conducted in line with the NICE Guide to the Methods of Technology Appraisal (2013) [61]. In particular, an NHS and Personal Social Services (PSS) perspective will be taken for costs, and health benefits will be quantified using QALYs.

The primary analysis will be an economic evaluation alongside the clinical trial, and will use the 1-year follow-up period of the trial to estimate expected 1-year costs and QALYs for the intervention and control groups. QALYs will be estimated using the EuroQol (5-level) questionnaire reported at baseline, 6 months and 12 months. The EuroQol will be valued using population tariff values to estimate EQ-5D-5 L scores. Although these are not yet published, they are expected to be available during 2013. QALYs will be estimated using the trapezium rule to calculate the area under the curve.

NHS resource use will be measured for each participant between baseline and final follow-up. This will include all medication costs, visits to health services and any social care and community support. Medical costs will be taken from the trial medication records, and other NHS and resources used will be self-reported using the widely used and validated Client Service Receipt Inventory (CSRI) questionnaire [54]. Unnecessary questions in the CSRI will be removed to reduce the burden for patients; however, questions relating to personal costs incurred and time off work will be retained for a sensitivity analysis.

The estimate of cost-effectiveness will be reported as the incremental cost-effectiveness ratio (ICER). Patient variation in resource use and effectiveness will be captured by confidence intervals of the cost and outcome estimates separately. Due to the ratio property of the ICER, confidence intervals are less reliable and, therefore, bootstrapped estimates of the ICERs will be sampled to allow the probability of the intervention being cost-effective to be determined. This estimate of uncertainty will be reported using cost-effectiveness acceptability curves (CEACs), which report the probability that the intervention is cost-effective for any given level of willingness to pay.

A secondary analysis will be undertaken with a wider societal perspective. Personal costs and time off work will be included, as reported by patients using the CSRI questionnaire. Time off work will be valued as productivity losses using the Human Capital Method.

A potential tertiary analysis will include an extrapolation of the costs and benefits of the intervention, to allow a life-time estimate of expected costs and QALYs. Time to loss of efficacy of the intervention will be determined by a survival analysis of the within-trial data. This will be included in an established decision analytic model (The Sheffield RA Model) [62]. The model will determine the future treatment pathway for the patient populations once a switch from intensive DMARD therapy is estimated, including biologics if patients progress to severe RA.

## Discussion

TITRATE is intended to establish the clinical and cost-effectiveness of intensive treatment strategies in established RA patients with intermediate disease activity.

Three complex problems have come to light during our ongoing research into RA patients with intermediate disease linked to the TITRATE trial. The first of these is the potential heterogeneity of these patients. In some there is evidence of persisting inflammatory arthritis, with several swollen joints and an elevated ESR. In others the DAS28 mainly reflects high tender joint counts and high patient global assessments; these latter patients may have a pattern of fibromyalgic RA and they may not respond greatly to DMARDs [63]. There is insufficient information about whether or not fibromyalgic RA represents a distinct group of patients or whether it reflects higher pain scores in a minority of patients who otherwise have a similar clinical phenotype. More information is needed to resolve this question.

The second problem is whether remission is the optimal target, or if low disease activity is adequate. Although disability is minimised and QoL is maximised when patients achieve remission, more patients will achieve low disease activity and remission than remission alone. For this reason, it might be preferable to have a broader target which is achieved more often than the narrower target of remission. Secondary analyses will examine both remission and low disease activity states. However, further research is needed to identify the optimal treatment target, particularly in patients with established RA in whom some joint damage and disability may already have become irreversible.

The third potential problem is the marked variability in the training and experience of rheumatology nurses in England. Some rheumatology nurses are very experienced with high skill levels and knowledge. However, many have relatively little experience in the specialty. The extent of this variation reflects the absence of any agreed accredited national training programme for nurses. One complexity in assessing the impact of training is the relative propensity of healthcare professionals to learn and implement a new approach, such as the intensive management intervention we have described. Whilst experienced nurses may have the greatest knowledge and skills they may also be the least likely to adopt new approaches. It is likely that several nurse/practitioner-related factors are implicated when applying innovative management approaches to long-term disorders like RA.

Many RA patients have intermediate disease activity, so we anticipate that the results of the TITRATE trial will inform the NHS on how best to manage these patients and the benefits of delivering a more intensive management approach provided such an approach is found to be both effective and cost-effective.

Several other factors may influence interpretation of the trial results. One issue is that intensive management might have positive impacts on some secondary outcomes alone; for example, improving QoL without increasing remissions. As a number of secondary outcomes are being measured caution will be used in interpretation such findings. Another issue is that patients or clinicians may be reluctant to sufficiently increase therapy in the intensive management group or may give more intensive treatment to the usual care group. Finally, there is the potential impact of evaluating different response thresholds; defining remission using DAS28 involves dichotomising patients based on where they fall on a continuous line and it is possible that views will change on the optimal threshold for this division. Caution will be needed in interpreting the assessment of different thresholds in the final analysis.

### Trial status

Participants are currently being recruited. The first patient was enrolled in June 2014.

## Additional file

Additional file 1: SPIRIT Checklist figure. (PPTX 333 kb)

## Abbreviations

ACR: American College of Rheumatology
BIPQ: Brief Illness Perceptions Questionnaire
BMQ: Beliefs about Medicine Questionnaire
CRP: C-reactive protein
CSRI: Client Service Receipt Inventory
CTIMP: Clinical Trial of an Investigational Medicinal Product
DAS: Disease Activity Score
DMARD: Disease-modifying anti-rheumatic drug
EQ-5D-5L: EuroQoL 5-dimensional 5-level score
ESR: Erythrocyte sedimentation rate
GAD-7: Generalised Anxiety Disorder
GEE: Generalised estimating equations
HAQ: Health Assessment Questionnaire
IM: Intensive management
ITT: Intention-to-treat
MARS: Medication Adherence Rating Scale
MI: Multiple Imputations
NICE: National Institute of Clinical Excellence
non-CTIMP: non-Clinical Trial of an Investigational Medicinal Product
NRES: National Research Ethics Service
OR: Odds ratio
PHQ-9: Patient Health Questionnaire-9
QALY: Quality-adjusted life year
QoL: Quality of life
RA: Rheumatoid arthritis
RCT: Randomised controlled trial
SC: Standard care
SDAI: Simplified Disease Activity Index
TNFi: Tumour necrosis factor inhibitor
VAS: Visual Analogue Scale
