# Supplementary figures and images for: Does intensive management improve remission rates in patients with intermediate rheumatoid arthritis? (the TITRATE trial): study protocol for a randomised controlled trial
Source: Trials. 2017 Dec 8;18:591. doi: 10.1186/s13063-017-2330-8 (PMC5723045; doi:10.1186/s13063-017-2330-8)

## Slide 1
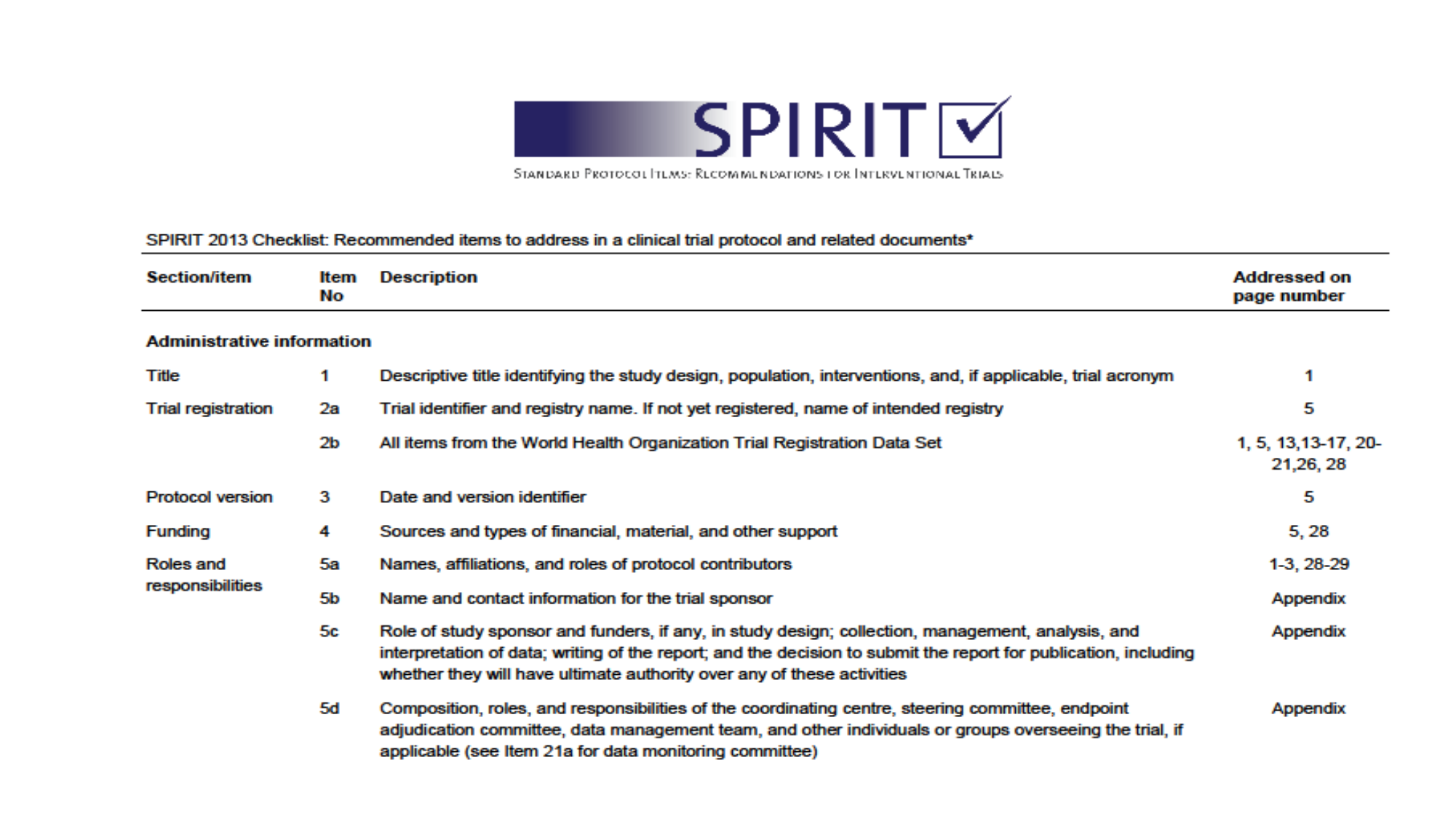

## Slide 2
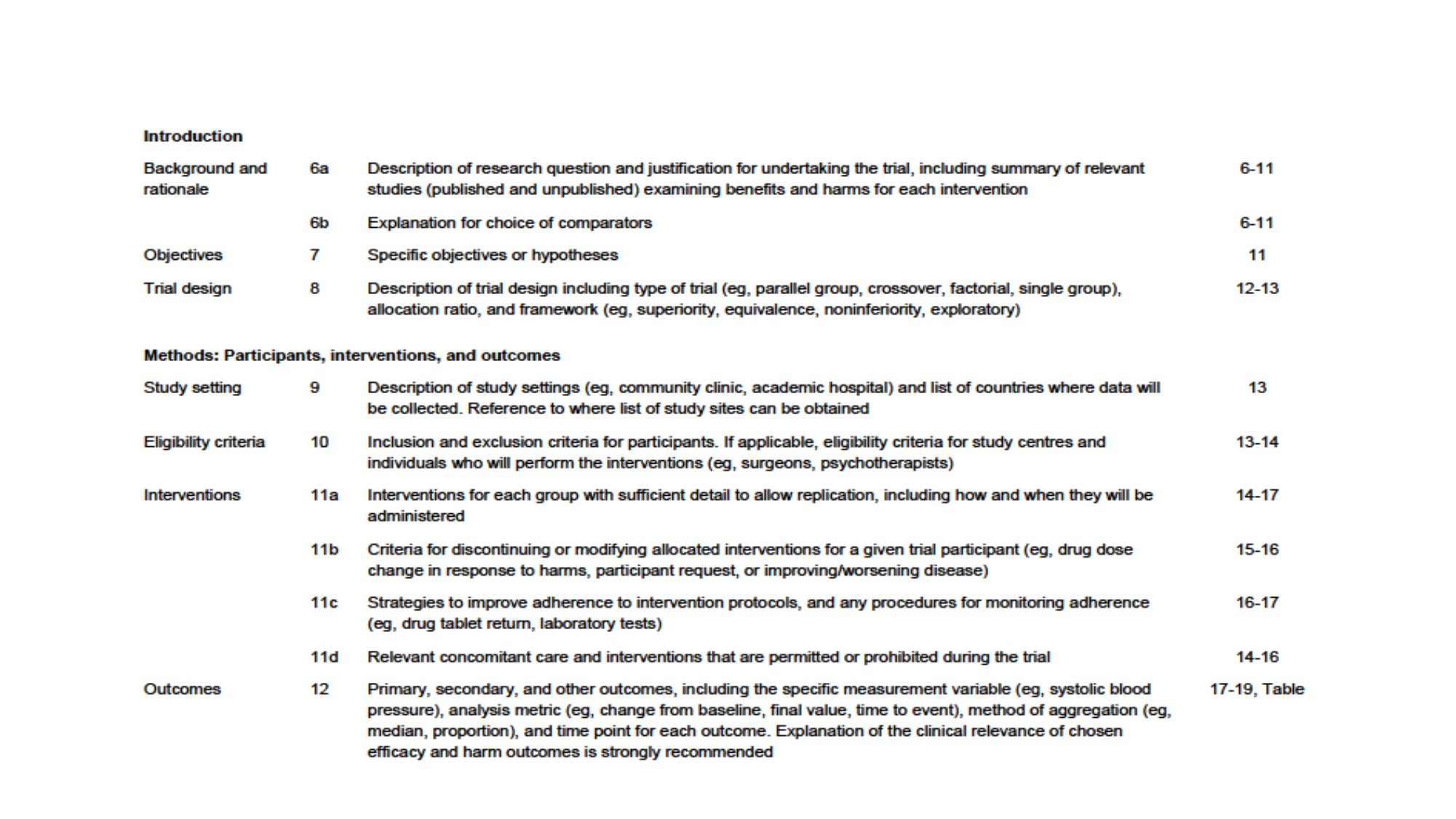

## Slide 3
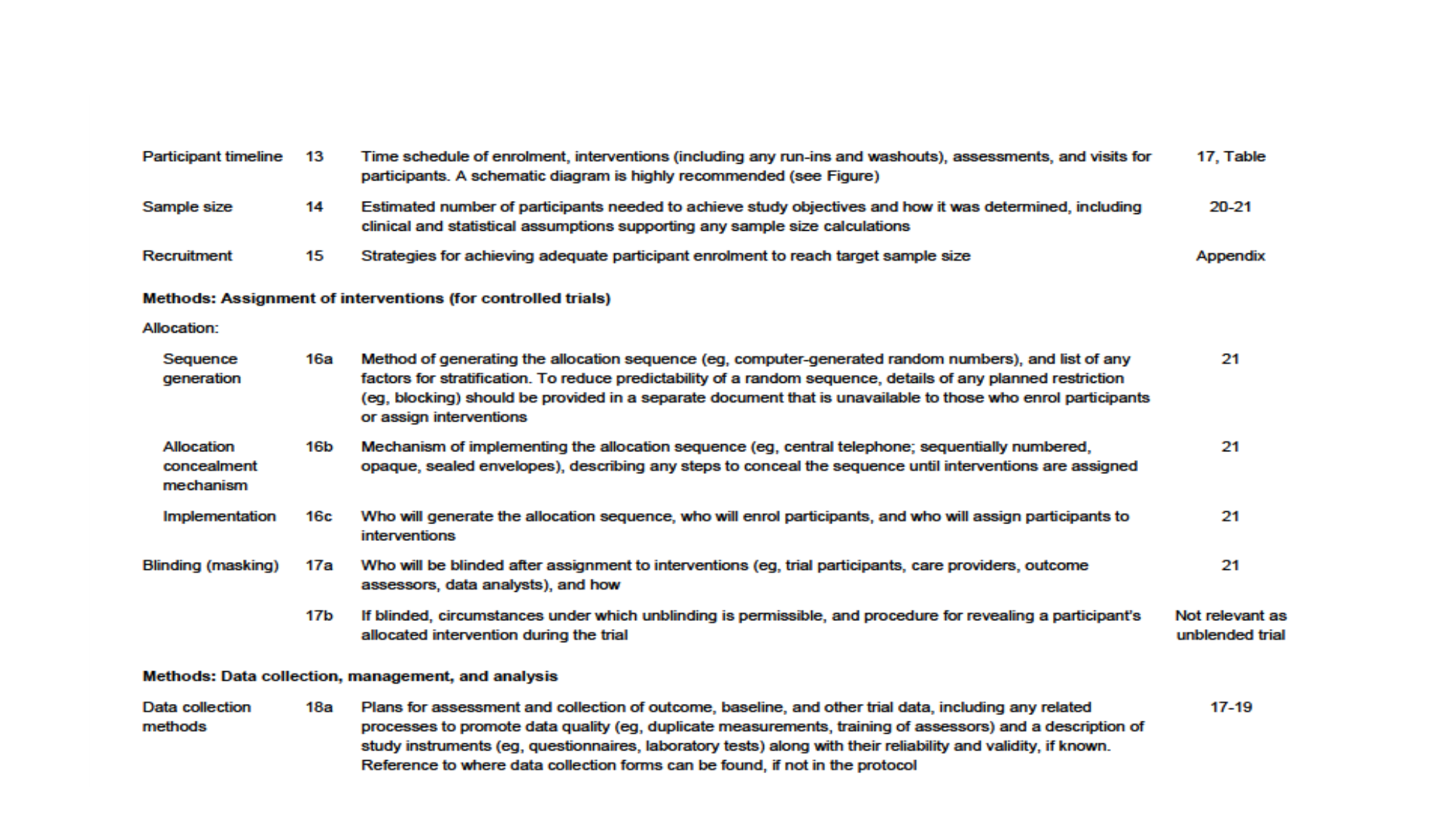

## Slide 4
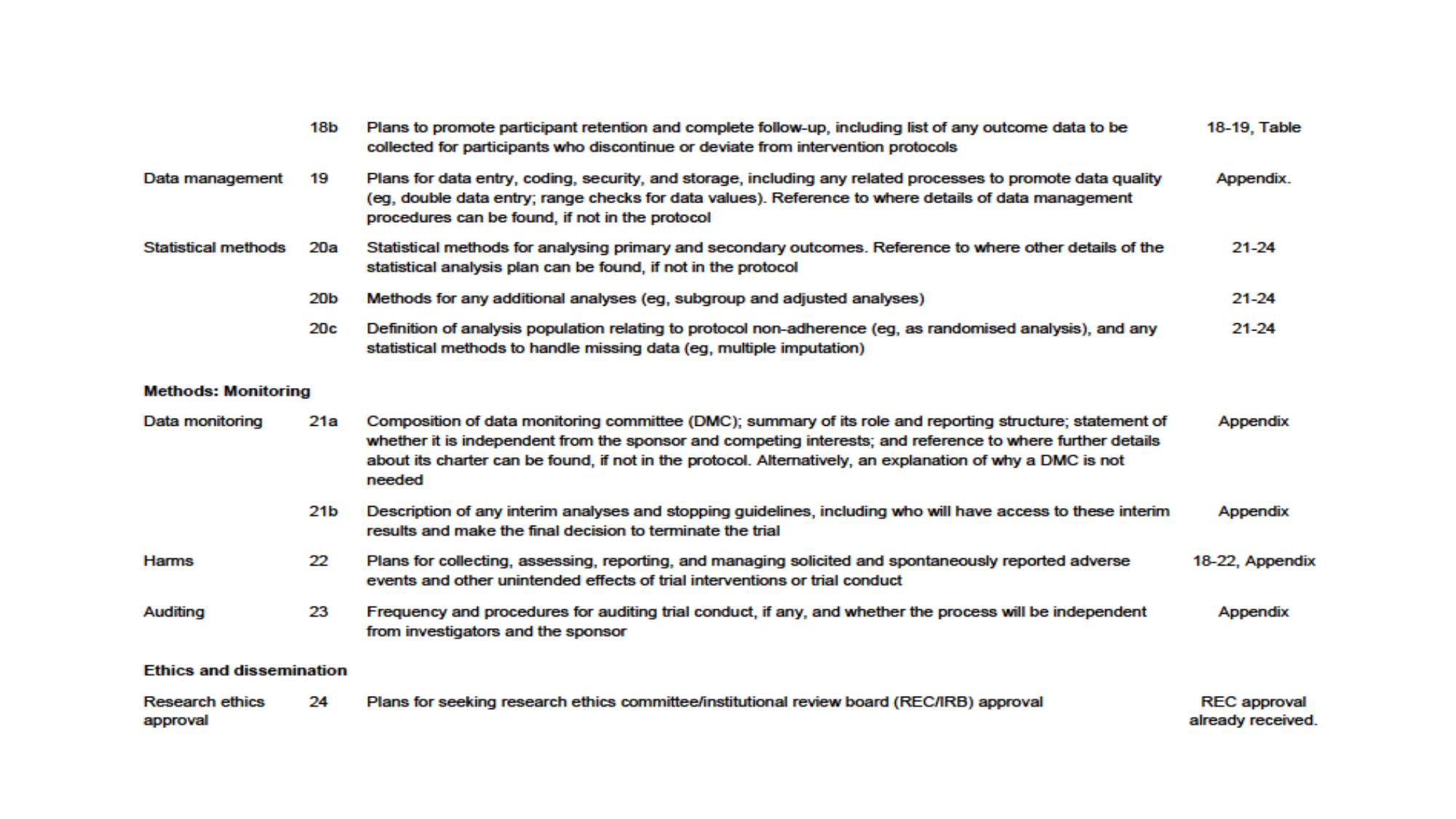

## Slide 5
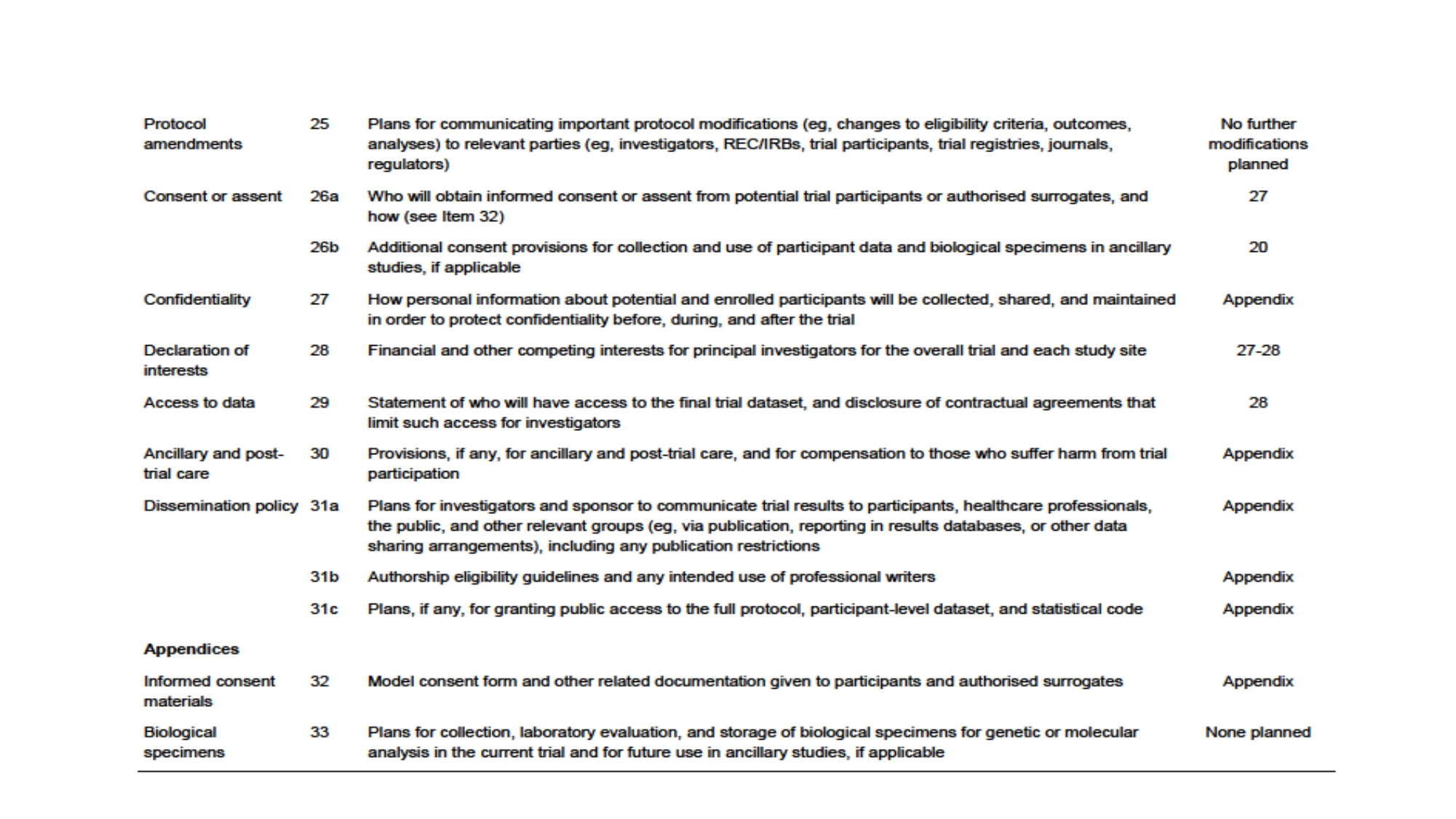

## Slide 6
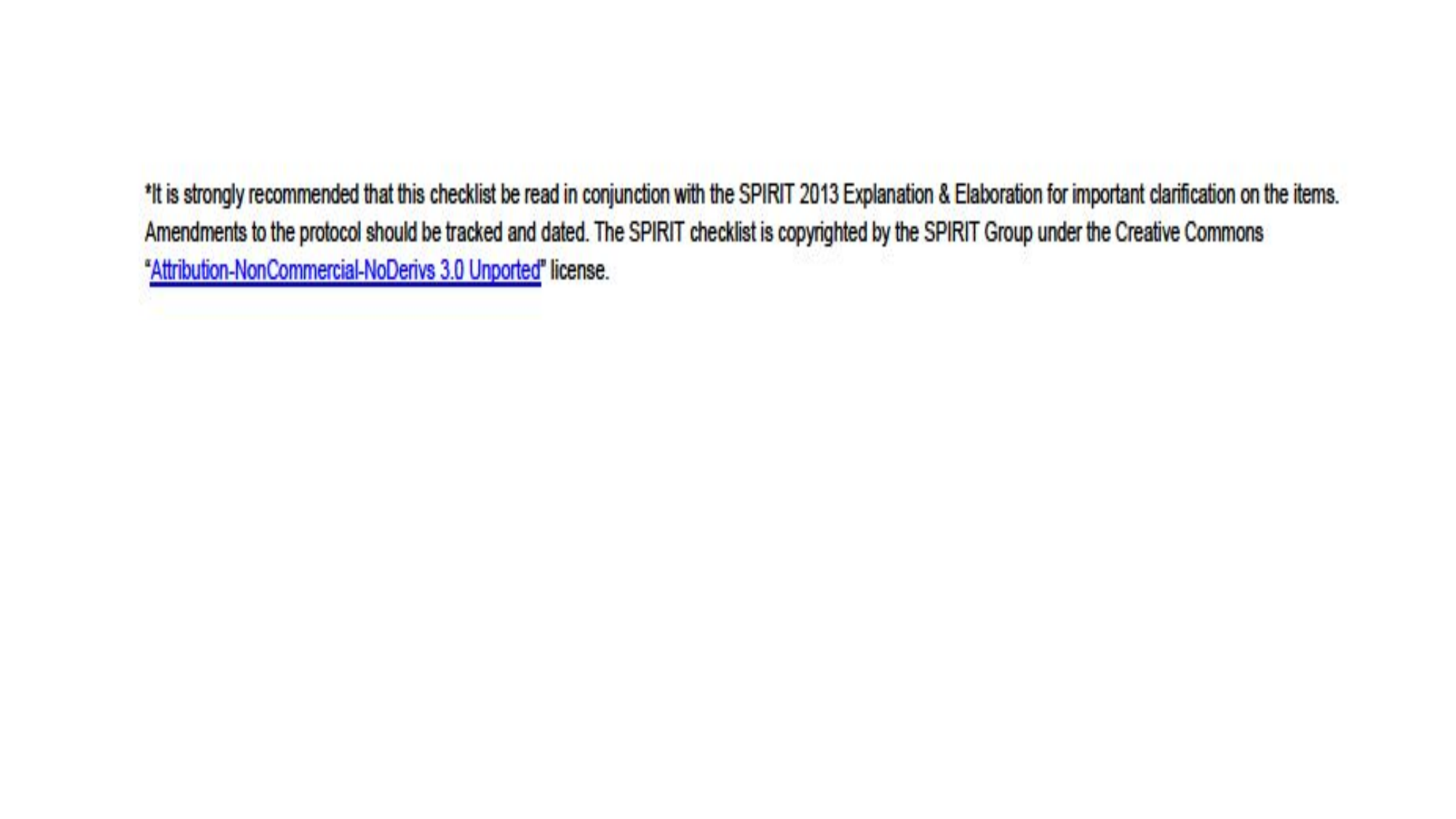

Supplement: Additional file 1: — SPIRIT Checklist figure. (PPTX 333 kb) [file 13063_2017_2330_MOESM1_ESM.pptx]
